# Agitation/excitement in male patients with treatment-resistant schizophrenia is associated with lower total cholesterol levels

**DOI:** 10.3389/fpsyt.2026.1917517

**Published:** 2026-08-26

**Authors:** Zhenkuo Li, Peng Xie, Cheng Yang, Lei Xia, Huanzhong Liu

**Affiliations:** 1 Department of Psychiatry, Dongguan Mental Health Center of Southern Medical University (Dongguan Seventh People’s Hospital), Dongguan, Guangdong, China; 2 Department of Psychiatry, The Fifth People’s Hospital of Xiangtan City, Xiangtan, Hunan, China; 3 Department of Psychiatry, Wuhu Hospital of Anding Hospital (The Fourth People’s Hospital of Wuhu), Wuhu, Anhui, China; 4 Department of Psychiatry, The Fourth Affiliated Hospital of Anhui Medical University, Hefei, Anhui, China; 5 Anhui Psychiatric Center, Anhui Medical University, Hefei, Anhui, China

**Keywords:** agitation/excitement, biological markers, lipid profile, male, PANSS-EC, total cholesterol, treatment-resistant schizophrenia

## Abstract

**Background:**

Treatment-Resistant Schizophrenia (TRS) in males with agitation/excitement poses a clinical challenge. The pathophysiological mechanisms of agitation/excitement in Male TRS remain unclear. This study explores these mechanisms and investigates potential biomarkers for agitation/excitement.

**Method:**

180 Male TRS patients and 100 healthy controls were enrolled. Psychiatric symptoms and agitation/excitement were assessed using the Positive and Negative Syndrome Scale 5-factor model (PANSS-5F) and the PANSS-Excited Component (PANSS-EC). Patients were divided into Male TRS with agitation/excitement (n=80) and without agitation/excitement (n=100). Demographic data and lipid profiles (triglycerides, total cholesterol, high-density lipoprotein, low-density lipoprotein) were collected for both groups. Statistical analyses assessed the relationship between lipid profiles and agitation/excitement.

**Results:**

A negative correlation was found between total cholesterol (TC) levels and agitation/excitement in Male TRS. Those with agitation/excitement had worse educational and marital status and more severe cognitive impairment. TC levels and hypercholesterolemia were higher in Male TRS compared to healthy controls.

**Conclusions:**

Lower TC levels in Male TRS with agitation/excitement are associated with higher agitation/excitement risk. TC may be an associated factor for agitation/excitement in this population.

**Clinical Trial Registration:**

https://chictr.org.cn, identifier ChiCTR2200063407.

## 1 Introduction

Schizophrenia is a disease characterized by multifaceted impairment of cognitive, affective, behavioral, and volitional activities, with a prolonged course and a high rate of disease recurrence, which can lead to varying degrees of impaired social functioning. As one of the ten most disabling disorders in the world, schizophrenia frequently results in severe adverse outcomes and imposes a heavy burden of illness on patients’ families (1). Agitation/Excitement is a common symptom of schizophrenia, with a much higher risk of agitation/excitement observed in this population compared to the general population (2). A study published in *The Lancet Psychiatry* found that the incidence of impulsive behaviors over a 5–10 years period was much higher in schizophrenia than in other psychiatric disorders (3). Moreover, impulsive and violent behaviors were much more frequent in men with schizophrenia than in women (4–6), often characterized by recklessness, cruelty, and overt perpetration (7). Agitation/Excitement in men with schizophrenia has attracted widespread social attention and has created a significant economic burden and safety risks for families and society (8).

Treatment-Resistant Schizophrenia (TRS) is a special group of patients with schizophrenia, accounting for approximately 20%-50% of patients (9). TRS is defined as the lack of response to a number of antipsychotic agents, which causes the patients to be actively symptomatic and to not gain symptom remission and functional recovery (10). Compared to non-refractory schizophrenia (non-TRS), TRS is more severe (11) and social functioning is more significantly impaired (12). Clozapine is currently recognized worldwide as an effective antipsychotic for TRS (13), and clozapine reduces impulsive behaviors in patients with schizophrenia (14), but only about 40%-70% of TRS patients respond effectively to clozapine (15), which leads to impulsive behaviors being more difficult to control in TRS patients. The study of impulsive behavior in patients with psychiatric disorders has always been a hot clinical research topic. However, so far the mechanism of impulsive behavior in patients with schizophrenia is still not completely clear, and even fewer studies have been conducted on impulsive behavior in TRS. Some studies have found a correlation between lipid profiles such as low-density lipoprotein, total cholesterol, and Triglyceride and impulsive, violent, and even suicidal behaviors in patients with schizophrenia (16–18). One main hypothesis of a possible biological mechanism which might explain the association is that low cholesterol levels in the central nervous system (CNS) may contribute to reduced transportation of serotonin through cholesterol-containing cell membranes. This may result in low levels of serotonin in the CNS and insufficient top-down control from the prefrontal cortex to the limbic structures of the brain, resulting in increased risk of affective and impulsive aggression (18–20). However, there is a lack of consistency in the findings of studies on correlates of impulsive behaviors in people with mental disorders, with several studies not finding similar positive results or gender differences in positive results (21). The inconsistency in the findings of these studies also suggests that there may be a more complex biological link between lipid profiles and impulsive behaviors that still needs to be explored and explained by many clinical studies. Male patients with refractory schizophrenia with concomitant agitation/excitement have received attention for their poorer outcomes and more difficult impulse control. Still, research evidence on the biological mechanisms of agitation/excitement in this population remains insufficient.

In summary, there has been increasing evidence suggesting that the lipid profile has the potential to be a biological marker of agitation/excitement in patients with psychiatric disorders, so the present study aimed to explore the biological relationship between lipid profile levels and agitation/excitement in male refractory schizophrenia patients with concomitant impulsivity, as well as between male refractory schizophrenia patients with and without concomitant impulsivity in terms of lipid profile differences. This study seeks to identify reliable biological markers of agitation/excitement that may assist in the identification, evaluation, and treatment of men with refractory schizophrenia who are at high risk for agitation/excitement in clinical practice.

## 2 Methods

### 2.1 Study design and participants

This was a single-center cross-sectional study. Our study included 180 Male Treatment-Resistant Schizophrenia (Male TRS, n=180) hospitalized in the Fifth People’s Hospital of Xiangtan City from September 1, 2022, to August 31, 2023, who met the following inclusion and exclusion criteria.

Inclusion criteria for Male TRS: 1. meet the diagnostic criteria for schizophrenia in the Diagnostic and Statistical Manual of Mental Disorders, 5th Edition (DSM-V). 2. male, aged 18–65 years, without major physical illness. 3. duration of disease <15 years. 4. TRS is defined as failure to respond to at least two adequate trials of different antipsychotic agents, each administered at chlorpromazine-equivalent doses of ≥600 mg/day for a minimum of 6 consecutive weeks, and the simultaneous presence of both (a) a severity score of ≥4 on the Clinical Global Impression–Severity (CGI-S) scale, and (b) a functional impairment score of ≤49 on the Functional Assessment for Comprehensive Treatment of Schizophrenia (FACT-Sz) or ≤50 on the Global Assessment of Functioning (GAF) scale (22). Exclusion criteria: 1. Severe physical illness and other organic brain diseases. 2. Comorbid substance abuse. 3. Comorbid mental retardation.

We referred to several clinical studies on agitation/excitement in patients with schizophrenia and used the definitions of agitation/excitement from these studies: Scores of ≥60 on the Positive and Negative Syndrome Scale 5-factor model (PANSS-5F) and ≥14 on the PANSS- Excited Component (PANSS-EC), with a score of ≥4 on at least one of the five items (23, 24). Male TRS (n=180) was categorized into Male Treatment-Resistant Schizophrenia with agitation/excitement (Male TRS with agitation/excitement, n=80) and Male Treatment-Resistant Schizophrenia without agitation/excitement (Male TRS without agitation/excitement, n=100).

This study also recruited 100 men who underwent a physical examination at the hospital’s health center during the same period as Healthy controls (HCs, n=100). General demographic information and blood test parameters of HCs were obtained from the hospital’s health screening system. HCs had to meet the following inclusion criteria as well as exclusion criteria. Inclusion criteria: (a) Male, no mental illness, and no family history of mental illness. (b) Be between 18 and 65 years of age. (c) Volunteer to participate in the study and sign an informed consent form. Exclusion criteria: (a) Psychoactive substance abusers. (b) Severe physical illness. (c) Comorbid mental retardation.

This study received approval from the Ethics Committee of the Fifth People’s Hospital of Xiangtan City (approval number: 2022003) and was conducted with no conflicts of interest, adhering strictly to the Declaration of Helsinki. Detailed information about the study was provided to all participants, who then voluntarily signed informed consent forms, ensuring their cooperation and the protection of their privacy rights throughout the research process. Clinical trial registration: https://chictr.org.cn, identifier: ChiCTR2200063407, registration date: 6 September 2022.

### 2.2 Outcome measures

Researchers collected general demographic information (Age, Education, Marital status, Employed Status, Household Registration, etc.) and general clinical information (Age of Onset, Duration of Illness, Number of Hospitalizations, etc.) from the Male TRS group (n=180) using a self-administered scale. The PANSS 5-factor model (PANSS-5F) was used to assess clinical psychiatric symptoms of patients. Blood samples from patients were collected between 06:00 and 07:00 AM after an overnight fast. Triglyceride (TG), total cholesterol (TC), high-density lipoprotein (HDL), and low-density lipoprotein (LDL) were detected. TG was measured by the GPO–PAP method. TC was measured by the CHOD–POD method. HDL-C and LDL-C were measured by the terminal method.

### 2.3 Primary outcomes

Agitation/Excitement was assessed by the PANSS-EC, TC lab results were obtained from blood samples drawn in the fasting state, and the PANSS-EC score and TC metrics were set as the primary endpoint. The PANSS-EC is a subscale of the PANSS, which has been extensively used to measure agitation/excitement in clinical pharmacotherapy trials (25, 26). The PANSS-EC consists of five individual PANSS items: hostility, uncooperativeness, impulsivity, tension, and excitability. The severity of each item is rated from 1 = nonexistent to 7 = extreme. Higher scores on the PANSS-EC indicate greater severity of agitation/excitement.TC is a biomarker of hotspots in the relationship between impulsivity and lipid profiles and has been used as a focal point of research in many studies (27), so this study also used TC as a primary observation.

### 2.4 Secondary outcomes

PANSS-5F, PANSS-Negative Component (PANSS-Ne), PANSS-Cognitive Component (PANSS-Co), PANSS-Depressive/Anxiety Component (PANSS-DA), PANSS- Positive Component (PANSS-Po) scores and other members of the lipid profile family (including TG, HDL, LDL, and hypercholesterolemia prevalence (28) were set as secondary outcomes. The PANSS-5F scores indicate overall psychiatric symptom severity, with higher scores indicating greater severity of the condition. Similarly, the PANSS-Ne, PANSS-Co, PANSS-DA, and PANSS-Po scores indicate the severity of the patient’s negative, cognitive impairment, anxiety-depression, and positive symptoms, respectively, with higher scores indicating a more severe condition. In studies of patients with schizophrenia with agitation/excitement, it has been shown that the PANSS-5F model can better characterize the patient’s condition in a multidimensional way (29–31), so we applied the PANSS-5F model to assess the psychiatric symptoms of the enrolled patients.

## 3 Statistical analysis

Data were analyzed using IBM SPSS Statistics 25.0 categorical variables were described by rate (%), and continuous variables were described by Mean ± SD or median (IQR) depending on whether they conformed to a normal distribution. Chi-square tests, independent samples t-tests, or Mann-Whitney U tests were performed between groups. In the Male TRS group, whether or not the impulses were accompanied was used as the dependent variable, the unaccompanied impulses group was assigned the value of 0 and the accompanied impulses group was assigned the value of 1. The results of these analyses were analyzed using logistic regression analyses for factors associated with impulsivity in the Male TRS group and calculated the odds ratio (OR) and 95% confidence interval (95% CI). Uncorrected models tested only demographic variables totaling 6 items (Age, Education, Marital status, Employed Status, Household Registration, Positive family history of schizophrenia). Model 1 tested demographic variables with clinical variables (Age of Onset, Duration of Illness, Number of Hospitalizations, olanzapine equivalent dose) totaling 10 items. Model 2 tested Model 1 + scale-related variables (PANSS-Po, PANSS-Ne, PANSS-Co, PANSS-DA) totaling 14 items and excluded PANSS-5F and PANSS-EC because grouping in the Male TRS was based on the PANSS-EC, which was also directly involved in the composition of the total score of the PANSS-5F. Model 3 tested Model 1 + Model 2 + lipid-related variables (TG, TC, HDL, LDL) totaling 18 items. All statistical results were considered statistically significant at *p* < 0. 05 (2-tailed).

## 4 Results

### 4.1 Comparison of general information and lipid profile levels in Healthy controls and Male TRS group

The mean age of the Male TRS group (42.52 ± 8.33) years and the mean age of the Healthy control group (43.32 ± 9.43) years were not statistically significant. No statistically significant differences were found in Employed Status or Household Registration between the Male TRS and HCs groups. The mean years of education of the Male TRS group (9.22 ± 1.85) years was significantly lower than that of the HCs group. The mean years of education in the TRS group (9.22 ± 1.85) was significantly lower than the mean years of education in the HCs group (11.89 ± 2.03), and the difference was statistically significant (*p* < 0.001). Marital status was statistically different between the two groups (*p* < 0.005). The married rate was significantly higher in HCs (83%) than in Male TRS (66.7%), and the single rate (5%) and the divorce rate (12%) were lower in HCs group than in Male TRS group (16.7%,16.7%), respectively. The TC level in the Male TRS group (4.41 ± 0.85) and prevalence of hypercholesterolemia (67.2%) were higher in the Male TRS group (3.17 ± 1.01, 34%) than in the HCs group (3.17 ± 1.01, 34%), respectively, and the differences were statistically significant (all *p* < 0.001). No statistical differences were found between TG, LDL, and HDL in the two groups. See **Table 1**.

**Table 1:** Comparison of socio-demographic information and clinical characteristics of the healthy control group and Male TRS group.

Variables	Healthy Controls (n=100)	Male TRS (n=180)	Statistics	
			X^2/^t/Z	*P*
Age (year)	43.32 ± 9.43	42.52 ± 8.33	0.71	0.48
Education (year)	11.89 ± 2.03	9.22 ± 1.85	10.88	<0.001
Marital status			7.8	<0.005
Married	83(83%)	120(66.7%)		
Single	5(5%)	30(16.7%)		
Divorced	12(12%)	30(16.7%)		
Employed Status	77(77%)	130(72.2%)	0.53	0.47
Household Registration			0.02	0.89
Local Resident	55(55%)	96(53.3%)		
Non-local Resident	45(45%)	84(46.7%)		
Positive family history of schizophrenia		16(8.9%)		
Age of Onset (years)		31.40 ± 5.062		
Duration of Illness(months)		86.79 ± 43.18		
Number of Hospitalizations		5.04 ± 1.50		
Olanzapine equivalent dose (mg)		11.29 ± 4.75		
PANSS-5F		96.77 ± 10.55		
PANSS-EC		13.84 ± 3.61		
PANSS-Po		20.01 ± 1.81		
PANSS-Ne		18.87 ± 2.07		
PANSS-Co		18.07 ± 1.25		
PANSS-DA		20.98 ± 3.11		
Prevalence of hypercholesterolaemia(%)	34(34%)	121(67.2%)	27.38	<0.001
TG(mmol/L)	0.99 ± 0.45	1.09 ± 0.30	-1.77	0.077
TC(mmol/L)	3.17 ± 1.01	4.41 ± 0.85	-10.4	<0.001
HDL(mmol/L)	1.24 ± 0.21	1.21 ± 0.32	0.94	0.346
LDL(mmol/L)	2.77 ± 6.42	2.97 ± 8.65	-0.22	0.826

n, sample size; *P*, probability; %,percent; PANSS-5F, Positive and Negative Syndrome Scale five-factor; PANSS-EC, Positive and Negative Syndrome Scale-Excited Component; PANSS-Po, Positive and Negative Syndrome Scale-Positive Component; PANSS-Co, Positive and Negative Syndrome Scale-Cognitive Component; PANSS-DA, Positive and Negative Syndrome Scale-Depressive/Anxiety Component; TG, triglyceride; TC, total choles; HDL, high-density lipoprotein; LDL, low-density lipoprotein.

### 4.2 Comparison of general demographic data and general clinical data between Male TRS with agitation/excitement and Male TRS without agitation/excitement

No significant statistical differences were found between Male TRS with agitation/excitement and Male TRS without agitation/excitement in terms of Age, Education, Marital Status, Employment Status, and Household Registration. Additionally, there were no significant statistical differences between the two groups regarding Age of Onset, Duration of Illness, Number of Hospitalizations, Olanzapine Equivalent Dose, or Positive Family History of Schizophrenia. See **Table 2**.

**Table 2:** Comparison of sociodemographic information and clinical characteristics between Male TRS with agitation/excitement and Male TRS without agitation/excitement.

Variables	Male TRS without agitation/excitement (n=100)	Male TRS with agitation/excitement (n=80)	Statistics	
			X^2/^t/Z	P
Age (year)	42.04 ± 7.58	43.11 ± 9.18	-0.858	0.392
Education (year)	9.35 ± 1.77	9.05 ± 1.96	1.08	0.282
Marital status			0.349	0.84
Married	65(65%)	55(68.8%)		
Single	18(18%)	12(15%)		
Divorced	17(17%)	13(16.3%)		
Employed Status	70(70%)	60(75%)	0.554	0.457
Household Registration			0.04	0.841
Local Resident	54(54%)	42(52.5)		
Non-local Resident	46(46%)	38(47.5%)		
Positive family history of schizophrenia	10(10%)	6(7.5%)	0.343	0.558
Age of Onset (years)	31.54 ± 5.09	31.23 ± 5.06	0.414	0.679
Duration of Illness (months)	87.65 ± 42.72	85.71 ± 44.01	0.298	0.766
Number of Hospitalizations	5.19 ± 1.43	4.86 ± 1.57	1.462	0.145
Olanzapine equivalent dose (mg)	8.61 ± 3.15	8.39 ± 1.58	0.531	0.595
PANSS-5F	92.12 ± 11.33	102.59 ± 5.47	-7.584	<0.001
PANSS-EC	10.97 ± 1.35	17.43 ± 1.97	-26.031	<0.001
PANSS-Po	19.95 ± 1.83	20.08 ± 1.80	-0.458	0.647
PANSS-Ne	18.93 ± 2.09	18.79 ± 2.07	0.457	0.648
PANSS-Co	18.19 ± 1.25	17.93 ± 1.23	1.421	0.157
PANSS-DA	21.03 ± 3.13	20.91 ± 3.12	0.251	0.802
TG	1.10 ± 0.29	1.08 ± 0.31	0.521	0.603
TC	4.68 ± 0.78	4.08 ± 0.82	4.963	<0.001
HDL	1.21 ± 0.34	1.22 ± 0.28	-0.309	0.757
LDL	2.29 ± 0.66	2.35 ± 0.72	-0.576	0.566

n, sample size; *P*, probability; %, percent; PANSS-5F, Positive and Negative Syndrome Scale five-factor; PANSS-EC, Positive and Negative Syndrome Scale-Excited Component; PANSS-Po, Positive and Negative Syndrome Scale-Positive Component; PANSS-Co, Positive and Negative Syndrome Scale-Cognitive Component; PANSS-DA, Positive and Negative Syndrome Scale-Depressive/Anxiety Component; TG, triglyceride; TC, total choles; HDL, high-density lipoprotein; LDL, low-density lipoprotein.

### 4.3 Comparison of the PANSS-5F model between Male TRS with agitation/excitement and Male TRS without agitation/excitement

No statistical differences were found between Male TRS with agitation/excitement and Male TRS without agitation/excitement in terms of PANSS-Ne, PANSS-Po, PANSS-DA, and PANSS-Co. Compared with Male TRS without agitation/excitement, Male TRS with agitation/excitement had higher PANSS-5F and PANSS-EC scores, and the difference was statistically significant (all *p* < 0.001).

### 4.4 Comparison of lipid profile levels between Male TRS with agitation/excitement and Male TRS without agitation/excitement

No statistical differences were found between Male TRS with agitation/excitement and Male TRS without agitation/excitement in terms of LDL, HDL, and TG. Compared with Male TRS without agitation/excitement, Male TRS with agitation/excitement had lower TC levels, and the difference was statistically significant (*p* < 0.001).

### 4.5 Correlations between TC levels of Male TRS and factors of the PANSS-5F model

As can be seen in **Table 3**, Pearson correlation analysis in the Male TRS group showed a negative correlation between TC levels and PANSS-5F (r=-0.246, *p* = 0.001), PANSS-EC (r=-0.326, *p* < 0.001), and PANSS-Co (r=-0.203, *p* = 0.016). No significant correlations were found between TG, HDL, or LDL and the factors of the PANSS-5F model.

**Table 3:** Pearson correlation coefficients (r) between the PANSS-5F model and lipid profile in Male TRS.

Variables	PANSS-5F	PANSS-Ec	PANSS-Po	PANSS-Ne	PANSS-Co	PANSS-DA
TG	-0.079	-0.028	-0.084	-0.044	-0.001	0.186
TC	-0.246**	-0.326**	-0.008	0.022	-0.203**	-0.129
HDL	-0.06	0.023	-0.09	0.113	-0.072	0.103
LDL	0.011	-0.005	-0.049	0.023	0.103	-0.06

*P<0.05; **P<0.01; PANSS-5F, Positive and Negative Syndrome Scale five-factor; PANSS-EC, Positive and Negative Syndrome Scale-Excited Component; PANSS-Po, Positive and Negative Syndrome Scale-Positive Component; PANSS-Co, Positive and Negative Syndrome Scale-Cognitive Component; PANSS-DA, Positive and Negative Syndrome Scale-Depressive/Anxiety Component; TG, triglyceride; TC, total choles; HDL, high-density lipoprotein; LDL, low-density lipoprotein.

### 4.6 Factors associated with agitation/excitement

In a multi-model regression analysis based on binary logistic regression, we did not obtain positive results in the uncorrected model. In the 3 models, we found that the risk of agitation/excitement was lower with more hospitalizations (model 1: OR = 0.68, 95% Cl=0.47-0.98, P = 0.036; model 2: OR = 0.58, 95% Cl=0.35-0.86, P = 0.028; model 3: OR = 0.77, 95% Cl=0.61-0.99, P = 0.041). In addition, in Model 3, we found that the lower the level of TC, the higher the risk of agitation/excitement (OR = 0.47, 95% Cl=0.23-0.95, P = 0.023) (**Table 4**).

**Table 4:** Multi-model regression analysis of factors associated with agitation/excitement in the Male TRS OR (95% CI).

	Uncorrected model	Mode 1	Mode 2	Mode 3
Number of Hospitalizations		0.68(0.47-0.98)*	0.66(0.45-0.96)*	0.77(0.61-0.99)*
TC				0.47(0.23-0.95)*

*P<0.05; TC, total choles.

The uncorrected model included 6 variables: Age + Education + Marital status + Employed Status + Household Registration + Positive family history of schizophrenia.

Model 1 included 10 variables: uncorrected model + Age of Onset + Duration of Illness + Number of Hospitalizations + olanzapine equivalent dose.

Model 2 included 14 variables: model 1 + PANSS-Po + PANSS-Ne + PANSS-Co + PANSS-DA.

Model 3 included Model 2 + TG + TC + HDL + LDL.

## 5 Discussion

Our findings confirmed our primary hypothesis, and the main findings were as follows. First, compared to healthy controls, Male TRS had poorer mean education and marital status, and Male TRS had higher TC levels. Second, compared to Male TRS without agitation/excitement, Male TRS with agitation/excitement had higher PANSS-5F and PANSS-EC scores, and lower TC levels. Third, in Male TRS, TC levels were negatively correlated with PANSS-5F, PANSS-EC, and PANSS-Co, respectively. And more hospitalizations were found to be a protective factor for agitation/excitement and lower TC levels were found to be a risk factor for agitation/excitement in the multi-model regression analysis of binary logistic regression.

In our study, Male TRS were found to be less educated than the healthy population and had similarly poorer marital status than the healthy population (**Table 1**). Several review studies have mentioned that the schizophrenia population is generally poorly educated (32, 33), but none of these studies have delved into whether there are differences in educational attainment between subtypes of schizophrenia, such as TRS. The current state of poor educational attainment in Male TRS may be related to a variety of factors. One study found that women with schizophrenia typically have a later age of onset than men and that positive and negative symptoms contribute to decreased learning ability and social regression in both male and female patients (34). Early onset of schizophrenia and exposure to psychotic symptoms puts male patients’ schooling at risk, coupled with a more severe risk of violence than female patients (4–6) and disease characteristics of poorer efficacy of TRS (11), all of which may contribute to the inability of schools to accept these male patients and thus deprive them of the opportunity to continue their education. So it is not difficult to explain why Male TRS have a lower level of education, especially compared to the healthy population. In addition, a comparison of general demographic information revealed that the marital status of Male TRS was worse compared to healthy controls (**Table 1**). The above potential influences on the differences in educational attainment between the two groups may equally influence their marital status. The higher risk of violence, lower social functioning, and especially poorer clinical outcomes in refractory schizophrenia in men with schizophrenia are all undesirable factors that may have a hindering effect on the choice of spouse in Male TRS. A review study described that the marital status of patients with schizophrenia is often dismal, and even those who form families have difficulty in obtaining family support from the couple relationship which eventually leads to marital breakdown (35). More recently, a study from China found that stigma was a serious problem for people with mental disorders and their family relationships and that they were almost reluctant to disclose information about their illnesses because of stigma (36). Stigma about mental illness is indeed prevalent in China (37), and excessive stigma may cause people with mental illness to avoid contact with the different sex and even lose the opportunity to form a family, which may be an important factor contributing to the poorer marital status of people with Male TRS than the healthy population. In **Table 1** we also found a statistically significant difference in the prevalence of hypercholesterolemia and TC levels between Male TRS and the healthy population, with Male TRS having a much higher prevalence of hypercholesterolemia and TC levels than the healthy population. Regarding these two findings, they are consistent with the conclusions of some studies (38, 39), which have pointed out that long-term use of antipsychotics can cause disorders of glucose-lipid metabolism and even the development of metabolic syndrome, so it is not difficult to explain the fact that Male TRS has a higher prevalence of hypercholesterolemia and TC levels.

Compared with Male TRS without agitation/excitement, our study found that Male TRS with agitation/excitement had higher PANSS-5F and PANSS-EC scores (**Table 2**). We used the PANSS-EC definition of agitation/excitement to group the included Male TRS, so Male TRS with agitation/excitement had higher PANSS-EC scores as a result of the grouping requirement. A cross-sectional study by the team of Yi et al. exploring the clinical characteristics of schizophrenics with violence in China (40) found that patients in the violent group had higher total PANSS scores than those in the nonviolent group, which is consistent with our findings and implies that the population of patients with agitation/excitement has more severe psychiatric symptoms. Yi’s study also used the PANSS-5F model to assess patients’ psychiatric symptoms and also found that the violent group had higher PANSS-EC scores and PANSS-Po scores, but did not find Male TRS with agitation/excitement to have higher PANSS-Po scores in our study. In contrast to our study, Yi’s study used a self-administered questionnaire to define violent behavior, which lacked quantifiable indicators, and the definition of “violence” was not comprehensive enough, ignoring verbal violence and violence against oneself, such as self-inflicted suicide. It is possible that the difference between Yi’s team’s definition of “violence” and the definition of “impulsivity” in our study influenced the grouping results and led to partially different positive results in the PANSS-5F model.

The relationship between lipid profile levels and impulsivity has been a hot topic, and many studies have been based on the classic cholesterol-serotonin attack hypothesis proposed by Kaplan et al. that low levels of TC lead to attack by altering the serotonergic system (16, 18). In **Table 2**, it can be found that the TC level of Male TRS with agitation/excitement is significantly lower than that of Male TRS without agitation/excitement, and a negative correlation between TC level and PANSS-EC scores was found in Male TRS (**Table 3**), a finding that suggests that there is an underlying biological link, with lower TC levels potentially implying higher agitation/excitement. A cross-sectional study by Kavoor (41) et al. found a negative correlation between TC levels and severity of impulsivity, a finding that is consistent with the findings of our study, in addition, they found a similarly negative correlation between TC levels and propensity for suicidal impulsivity. Unlike our study, Kavoor’s team included drug-naïve/drug-free patients of schizophrenia, whereas our study was conducted on TRS patients on long-term antipsychotic medications or even multi-drug combinations, and similar findings were obtained in different study populations, suggesting that the relationship between impulsivity and TC levels may persist and persist throughout a schizophrenic patient’s illness, perhaps having little to do with whether or not they have been treated with antipsychotic medication. Studies by Roaldset (18) and Hillbrand (42) likewise came to similar conclusions, verifying the relationship between TC and impulsivity. In addition, a relationship between low TC and high risk of impulsivity has also been found in violent offenders without mental disorders (43, 44), and this research evidence suggests that the relationship between lipid profiles and impulsivity is not limited to mentally challenged populations, but also exists in impulsive healthy populations, particularly violent offenders. This may help to identify potentially high-risk violent populations at an early stage and reduce the incidence of violent crime. There are also prospective studies confirming the negative correlation between TC levels and impulsive behavior in patients with mental disorders (16). Lower TC implying a high risk of suicidal impulsivity has been found not only in the field of schizophrenia research but also in studies related to suicidal impulsivity in patients with major depression (45). Suicidal ideation and violent suicidal behavior were found to correlate with TC and leptin levels in a study on suicide attempts in patients with mental disorders (46). It has been shown that there is an interaction between the leptinergic and serotonergic systems in the CNS (47), and leptin has been observed to stimulate serotonin turnover (48). In turn, levels of TC in the central nervous system can influence the metabolism of the serotonergic system to induce impulsive and aggressive behaviors (20), so there appears to be an even more complex biological connection between TC, leptin, and serotonin, but research on the relationship between the three is lacking. Perhaps it is because of this complex interplay of influences that some studies have come to inconsistent conclusions. A study by Eriksen et al. found no significant association between TC levels and impulsivity, but there was a significant negative correlation between HDL levels and the severity of impulsivity during hospitalization and even follow-up, and this relationship was only found in male patients with acute-phase psychiatric disorders (17). Unlike the results of Eriksen et al.’s study, we did not find a correlational relationship between impulsivity and HDL, although our study included the same all-male patients and the difference in sample size could be a potential factor for the inconsistent results. In addition, Eriksen’s study included almost all acute-onset psychiatric disorders, including schizophrenia, bipolar disorder, depressive disorders, and even personality disorders, and the difference in sample size may have also contributed to the variability in results. Nevertheless, some studies report no association between TC and agitation/excitement (49).

In **Table 3**, it can be seen that there is a negative correlation between TC levels and PANSS-Co scores in Male TRS, which implies that patients with lower TC levels may have more severely impaired cognitive function. Similar conclusions were reached in a clinical study from China on cognitive functioning in patients with chronic schizophrenia, which found that elevated serum TC levels may be associated with improved cognitive functioning, especially that of immediate memory (50). Pang’s study (51) found that higher TC levels in older adults >60 years of age were protective of cognitive function in older women, an association that was not found in older men. Pang’s findings also suggest that there may be age and gender differences in the relationship between TC levels and cognitive function. Thorvaldsson’s study (52) similarly found an association between lower TC levels were associated with poorer cognitive function, and those with a significant trend of declining TC levels over time had a similarly prominent rate of cognitive decline and were more likely to develop dementia. In addition, Huan’s study (53) found that elevated serum TC may be a potential factor in preventing or mitigating cognitive dysfunction within the normal range of serum TC in a population of healthy older adults, but there were racial differences in this association. The findings of the above studies support our findings, but there is a lack of consistency in the findings of current clinical studies on the relationship between TC and cognitive function. For example, both research teams of Zhao (54) and Jia (55) found higher TC levels to be a risk factor for cognitive impairment. In addition to this, several clinical studies have explored the relationship between cognitive deficits and impulsive behavior in patients with psychiatric disorders. Ahmed et al. found that schizophrenic patients with violent offenses showed more severe impairments in most of the cognitive domains, and Cognitive deficits increase the risk of impulsive aggression in schizophrenia via inefficient regulation of negative affective states (56). A study from China found (40) that patients with violent behavior had lower RBANS language, semantic fluency, and total subscale scores. These studies verified that psychotic patients with impulsive behavior have worse cognitive functioning, and laterally support the negative correlation between serum TC and agitation/excitement. Perhaps cognitive impairment plays a mediating role between serum TC and agitation/excitement, but this still needs to be verified by clinical studies, and our next study will also focus on this. Furthermore, in **Table 3**, a negative correlation was found between PANSS-5F scores and serum TC, implying that Male TRS with lower levels of serum TC had more severe psychiatric symptoms. On this point, the study found that it is not difficult to explain that more severe cognitive impairment and agitation/excitement are a reflection of the severity of psychiatric symptoms. In addition, the relationship between the positive results in **Table 3** can be synthesized and analyzed to conclude that PANSS-EC and PANSS-Co may be the main reason for the negative correlation between PANSS-5F total score and TC.

A multi-model regression analysis of Male TRS found that a higher number of hospitalizations implied a lower risk of agitation/excitement in all three models (**Table 4**). This finding may be related to the unbalanced allocation of healthcare resources and inadequate community psychiatric rehabilitation systems in developing countries. The lack of timely access to quality community-based psychiatric rehabilitation services for discharged patients with mental disorders, coupled with the fact that the condition of TRS patients is more difficult to control, contributes to the high rate of relapse and recurrent hospitalization of patients with schizophrenia. One study found an extremely high risk of re-hospitalization in the first 60 days after discharge (57). Therefore, timely hospitalization during acute episodes of psychiatric symptoms may be the most effective way for patients with mental disorders in developing countries to receive specialized psychiatric care. More hospitalizations mean more healthcare resources are available, and systematic antipsychotic treatment will inevitably reduce the severity of psychiatric symptoms, especially agitation/excitement, which is more easily observed clinically. However, repeated hospitalization imposes a heavy economic burden on society and also takes up a large amount of medical resources (58), so it is particularly important to establish a comprehensive community-based psychiatric rehabilitation system (59, 60). In addition, lower TC levels can be found as a risk factor for agitation/excitement in **Table 4**, implying that the risk of agitation/excitement is more severe in Male TRS with low TC levels. Regarding this point of conclusion, the correlation relationship between lipid profile and agitation/excitement has been validated in **Table 3**. In addition, the inclusion of 18 variables in Model 3 for binary logistic regression analysis yielded positive results that shed even more light on the biological link between serum TC levels and patient agitation/excitement.

Returning to our findings and combining them with the previously listed studies that are consistent with our findings, we can prove the negative correlation between TC and agitation/excitement in Male TRS. However, we cannot ignore the studies that are inconsistent with our conclusions and have to pay more attention to them, because these inconsistent conclusions also reflect a more complex relationship between lipid profile and agitation/excitement from the side. This relationship may not be fully explained by the simple cholesterol-serotonin attack hypothesis, and this relationship may involve more complex metabolic and transport mechanisms, as well as more biological substances, such as leptin. In addition, we found that some of the existing studies on the correlation between lipid profiles and agitation/excitement included mixed samples, and the sample populations often included multiple psychiatric disorders, which suggests that less stringent sample inclusion and exclusion criteria may also have had an impact on the reliability and consistency of the findings. Therefore, more scientific, rigorous, and comprehensive studies are still needed to further substantiate the follow-up.

In this study, there are some limitations: 1) Sample Size and Generalizability: The study’s small sample size and single-center design limit the generalizability of the findings. 2) Gender Considerations: The trial exclusively included male patients with schizophrenia, overlooking potential gender differences in agitation/excitement. 3) Assessment Tools: Reliance on a single psychiatric assessment scale may not fully capture the complexity of the clinical symptoms. 4) Multiple comparisons: The authors performed numerous t-tests, correlation analyses, and logistic regressions without any correction for multiple testing (e.g., FDR or Bonferroni). This increases the risk of false-positive findings and should be explicitly acknowledged. 5) Unmeasured confounders: The study did not control for smoking status, body mass index (BMI), or history of lipid-lowering medication use—all of which are well-known confounders affecting lipid profiles. Adjustment solely for age and education is insufficient. 6) Inability to infer causality: Given the cross-sectional design, the study cannot distinguish whether low TC leads to agitation/excitement, or conversely, whether impulsive states are accompanied by reduced dietary intake or metabolic changes leading to low TC. These limitations suggest the need for further research with a more reasonable experimental design, including larger, multi-center trials that involve both male and female patients and utilize a broader range of neurophysiological markers and psychiatric assessment tools.

## 6 Conclusion

Our cross-sectional findings suggest that serum TC level is negatively associated with agitation/excitement scores in male TRS patients. This association warrants further validation in longitudinal cohorts to assess its potential utility as a risk stratification biomarker. The causal direction and underlying mechanisms remain to be elucidated.

## Data Availability

The raw data supporting the conclusions of this article will be made available by the authors, without undue reservation.
